# Stability and Instability of Subjective Well-Being in the Transition from Adolescence to Young Adulthood: Longitudinal Evidence from 20991 Young Australians

**DOI:** 10.1371/journal.pone.0156399

**Published:** 2016-05-27

**Authors:** Xidan Chen, Andrew Page

**Affiliations:** Centre for Health Research, School of Medicine, Western Sydney University, Sydney, New South Wales, Australia; University of New South Wales, AUSTRALIA

## Abstract

**Purpose:**

This study assessed the long-term stability and instability of subjective well-being during post-school transition (i.e., transition from adolescence to young adulthood) and evaluated the determinants of transition stability.

**Methods:**

Using two cohorts from a national representative longitudinal study, the Longitudinal Study of Australian Youth (N = 20991), latent profile analysis and latent transition analysis were conducted to examine transition patterns among subjective well-being profiles for youth from age 17 to 25. Multinomial logistic regressions were conducted to evaluate whether key socio-demographic variables were associated with transition stability.

**Results:**

We identified: (1) three subjective well-being profiles: Low (30%), Moderate (50%), and High (20%); and (2) three major transition patterns among these subjective well-being profiles: stable, partially-stable, and unstable. The majority of youth had stable transition patterns during the transition from adolescence to adulthood. A large percentage of youth (52%) started low in subjective well-being profile and remained in the low subjective-wellbeing profile. Our examination also revealed gender was the most pronounced indicator for transition stability during this time period, with males more likely to have unstable transition patterns than females.

**Conclusions:**

Results suggest that different subjective well-being status and transition patterns can be identified in the post-high school transition to adulthood, including unstable transitions. By targeting those groups more vulnerable to transition, mental health promotion and interventions may be delivered more effectively.

## Introduction

Subjective well-being represents people’s self-evaluations of their life including cognitive evaluation such as life satisfaction and affective evaluation such as sadness or joy [1]. It has been recognized as an important indicator for health. For example, increasing evidence supports the link between higher subjective well-being and better health such as adaptation ability [2], mental health [3], and psychosocial functioning [4] for youth and adolescents. In contrast, lower subjective well-being has been linked with negative health indicators such as adolescent substance abuse [5], and violent behaviours [6]. The link could vary over time or across age [7]. Examining whether subjective well-being is temporally stable has wide implications for health, economy and education policy, especially at sensitive transitional periods over the life-span, for example from adolescence to young adulthood [8]. In practice, a better understanding of the long-term stability of subjective well-being over the life-span could inform the timing of interventions for mental health promotion. Although temporal stability of subjective well-being has been studied extensively [9–11], stage change pattern has not been evaluated and the possible underlying sub-group change patterns have not been studied.

Mixed results have been reported regarding the long-term stability of subjective well-being, with previous studies showing substantial long-term stability [12,13] as well as instability [14,15]. Previous studies are predominantly based on cross-sectional data [16,17]. Stability therefore is not estimated directly rather it is inferred from age or different time points’ group mean differences in subjective well-being. Furthermore, time-scale often varies among these studies. With such cross-sectional data, it is therefore impossible to assess temporal relationships between life circumstances and subjective well-being at the individual-level.

More recent subjective well-being stability research have employed advanced analytical techniques [13,18], however, few analyses have been conducted to evaluate subjective well-being stability at the individual-level and in longitudinal study design. Among studies applying individual-level analysis [9,10], only global subjective well-being change patterns were assessed and stabilities of domain satisfactions (i.e. components of subjective well-being) were not directly evaluated. Previous studies have argued for the investigation of differential change patterns of domain satisfaction. Subjective well-being studies on age variation have found domain satisfaction change does not overlap with global subjective well-being change over time [17,19,20]. The differential change patterns of domain satisfaction indicate it is likely domain satisfaction might diverge from the mean level, and have higher unallocated instability than that found in global subjective well-being. Indeed, adopting Multidimensional Students’ Subjective Well-being Scale, Antaramian and Huebner [21] found the stability differences across different life domains.

The primary goal of this study is to evaluate the extent to which post-high school transition affects individual-level stability in subjective well-being. Post-high school transition is a life period that has not gained much attention in subjective well-being studies. It refers to the period when adolescents leave the compulsory education system, and start independent and divergent life paths (e.g., the start of a full-time job or vocational training) and is a marker of the change from adolescence to adulthood. A series of problems, challenges and life-adjustment situations are involved during this post-high school transition that may lead to the disruptions of social relationships, habits, and patterns of activities that affect long-term changes in various aspects of health and wellbeing [22–24]. As such, this study explores long-term stability of subjective well-being domains during post-high school transition applying individual-level analysis (latent transition analysis) on longitudinal data.

This study adopts a stage-sequential stability approach to explore the stage change pattern of subjective well-being profiles during post-high school transition. Subjective well-being profile describes an individual’s subjective well-being status in various life domains. Stage-sequential stability of subjective well-being profiles then describes an individual’s transition behavior at consecutive time points. The following specific research questions are addressed: First, is there a common set of subjective well-being profiles at each time points before, during and after post-high school transition? A common set of profiles supports the notion that individuals have differential subjective well-being statuses in various life domains. Second, how does an individual transit from one subjective well-being profile to another over time? If an individual’s transition probability of remaining in the same profile at two consecutive waves is high, and does not change over time, then subjective well-being can be considered stable. Third, if there are different transition patterns among profiles, is the stability of these transition patterns associated with particular socio-demographics?

## Materials and Methods

### Sample

Data were obtained from an ongoing annual national longitudinal project, the Longitudinal Surveys of Australia Youth (LSAY) [25]. LSAY is managed by the Australian Council for Educational Research (ACER) and the Commonwealth Department of Education, Science and Training (DEST), and is designed to track adolescents’ well-being during their transition into adulthood after they leave the compulsory education system [26**]**. Access to the LSAY was approved and made available by Australian Data Archive, and used in accordance with the LSAY privacy policy (https://www.ncver.edu.au/wps/portal/vetdataportal/restricted/privacy/). Personal information was anonymized by the ACER, and authors were not directly involved in data collection. Data are collected through phone interview up to 26 years of age. Questionnaires are similar each year with an emphasis on life circumstances change from school to post-high school education, vocational training and work. The present study was based on a combined analysis of two longitudinal samples comprising 7,378 participants from the cohort established in 2003 and 13,613 participants from the cohort established in 1995. Samples were selected only if participants completed subjective well-being measure starting from grade 12 (the last year for secondary education or high school), including 3 waves with a 2-year interval, and an age range from 17 to 25. Both cohorts have data missing on all three waves (2003 cohort *N*_missing_ = 1174; 1995 cohort *N*_missing_ = 3875). Cohort 2003 and Cohort 1995 have average of 38% and 48% missingness at item level and average yearly attrition rate of 9% and 6%, respectively. A list of sample characteristics is given in Table 1. High similarities share between these two cohorts offer the opportunity to cross-validate results.

**Table 1:** Sample Characteristics for Both Cohorts.

	Cohort 2003(N = 7378)	Cohort 1995(N = 13613)
**Female**	51%	51%
**Male**	49%	49%
**Age at Wave 1**	17.74 (*SD* = 0.27)	17.70 (*SD* = 0.47)
**Socio-Economic Class**		
*Lower*	29%	40%
*Middle*	43%	33%
*Upper*	28%	26%
**Status one year after post-high school transition**		
*University Study*	26%	58%
*Vocational Training*	26%	20%
*Employed*	61%	46%
Married or in a De Facto relationship*	3%	2%
**Status at Wave 3**		
*Completed at least one bachelor degree*	16%	14%
*Completed vocational training*	15%	10%
*Employed*	37%	28%
*Married or in a De Facto relationship*	12%	11%

*Note*: Social class for Cohort 2003 was converted based on the International Socio-Economic Index of Occupational Status (ISEI) [27]; Social class for Cohort 1995 was converted based on ANU 3 scale [28]; due to the missingness, overall percentage does not always equal to 100%;

*a De Factor relationship means a relationship as a couple living together on a genuine domestic basis.

### Measure

#### Subjective well-being scale

Subjective well-being scale used in LSAY is the Australian unity wellbeing index [29]. It was obtained from both cohorts. The scale contains 13 Likert-scale items (*1 = very unsatisfied to 5 = very satisfied*) for subjective well-being domains including work/study, career-prospects, future, living-standards, home-life, residence, independence, social-life, relationship, leisure, political and economic climate. The full questionnaire can be obtained from here (http://www.lsay.edu.au/publications/2297.html). Two Australian specific domains, political and economic climate were excluded from analyses as these two items were not available on all waves. Cronbach’s alphas for this subscale across waves ranges from 0.81 to 0.84 for Cohort 2003 and from 0.79 to 0.82 for Cohort 1995.

*Dimension reduction*. We conducted analyses to reduce dimensions of the 11-dimension subscale. There are two benefits of such manipulation. First, it reduces the computational loading of latent transition analysis. Second, as opposed to the previous studies combining it to a single score, this is a better use of information. Exploratory Structural Equation Modeling (ESEM) with Robust Weighted Least Squares estimator (WLSMV) for ordered categorical data [30] was used to explore how many latent dimensions were evident. ESEM incorporates Exploratory Factor Analysis into the Structure Equation Modeling construct, allows cross-loadings for measurement model, and thus yields generally a better fit [31]. From the 11 domains, three major dimensions across 3 time waves for both cohorts were identified: ‘achievement’, ‘family’, and ‘leisure’ (S1 Fig, S1 and S2 Tables) which are three major life domains for young adults [32–34]. Further, a longitudinal measurement invariance test over the three time waves was conducted to verify psychometric performance of current subjective well-being scale using this 3-factor ESEM model. Results show the measurement model as well as the structural relations are fully invariant across all waves (S3 Table, S2 Text), suggesting that the subjective well-being scale measures the same content over time. Factor scores of three dimensions were saved from the latent mean invariance model, and used as inputs for the following latent profile analysis and latent transition analysis.

#### Socio-demographic factors

Socio-demographic measures were obtained from Cohort 2003. Demographics include gender, indigenous, and immigration. Gender was measured on a two-point scale (1 = Male, 2 = Female). Indigenous Status was also measured on a two-point scale (0 = Non-Indigenous, 1 = Indigenous). Immigration status was measured as country of birth on a three-point scale (1 = Native students, 2 = First-Generation students, 3 = Non-native students). Family indicators include Social Economic Status (SES), sibling, and family structure. SES was measured as highest parental occupational status with four categories: blue collar low skilled, blue collar high skilled, white collar low skilled and white collar high skilled. Sibling was assessed by three measures: number of older siblings, number of younger siblings and number of same age siblings. We added up the numbers of three measures, then categorized it into a two-level variable: having-sibling *vs*. having-no-sibling. Family structure was measured by three categories: single parent family, mixed family and nuclear family. During post-high school transition, youth experience the major changes in their employment and education statuses, which further direct them onto different life paths [35]. This study therefore used employment and education status at each wave as life path indicators. Employment and education statuses are derived variables reported by LSAY developed from employment and educational data in year 2005, 2008 and 2011. Employment has two statuses: employed and unemployed. Education status was derived from full-time or part-time study statuses. We aggregated full-time or part-time study statuses as ‘studying’, others as ‘not-studying’.

### Analytical Strategy

All analyses were conducted using Mplus 7.1 with robust maximum likelihood estimator. Missingness was handled by the full information algorithms implemented in Mplus. Analyses were conducted on Cohort 2003 and cross-validated on Cohort 1995. We conducted latent transition analysis using the following modeling procedures [36]. First, Latent Profile Analysis (LPA) was explored at each time point in order to determine whether there was a common set of subjective well-being profiles (see S2 Fig for a theoretical model). LPA captures the relationship between a set of multivariate variables (usually continuous data) and a categorical latent variable. The categorical latent variable represents unobserved groups to which each participant could be assigned to according to their maximum likelihood probability. Following statistical indicators were considered to determine the best fitting model: the Akaike Information Criterion (AIC), the Bayesian Information Criterion (BIC), the Consistent Akaike Information Cariterion (CAIC), Adjusted BIC (ABIC), the entropy, the Lo, Mendell and Rubin likelihood ratio test (LMR), and the Bootstrap Likelihood Ratio Test (BLRT) (S1 Text). After the numbers of profiles were confirmed, Latent Transition Analysis (LTA) was conducted for the purpose of directly estimating subjective well-being stability. Latent transition analysis links the latent profile at one time point to a profile at the next time point by modeling changes in profile membership, and assesses stability over time in the form of movement between profiles. Finally, based on the LTA results, a series of multinomial logistic regressions were conducted to explore whether key socio-demographic factors were associated with transition stability.

## Results

### Identifying Transition Patterns among Subjective Well-Being Profiles

#### LPA results

**Fig 1:**
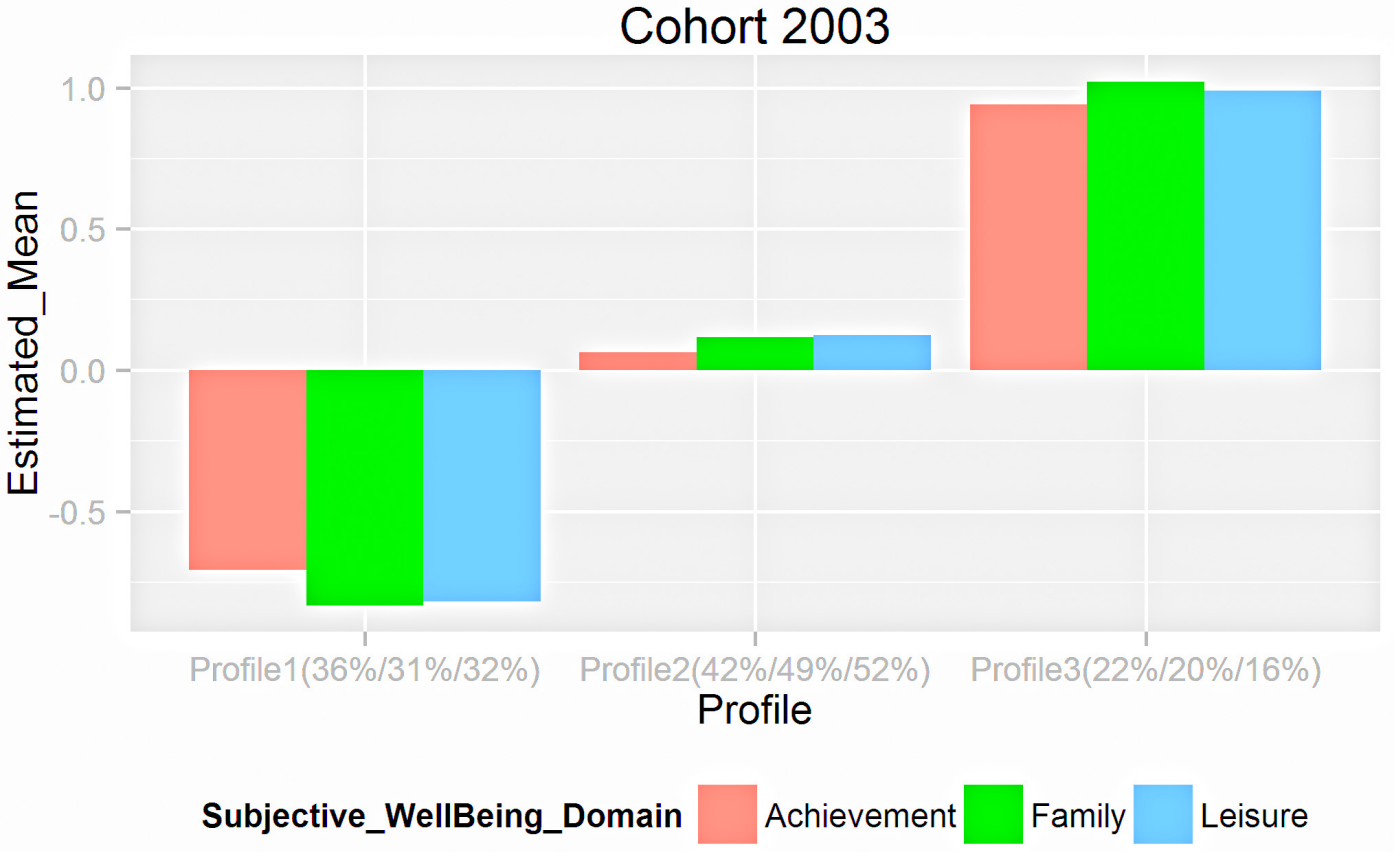
Subjective well-being profiles for Cohort 2003 across time from three-wave latent transition analyses. Fig presented here is based on raw scores. Percentages represent the proportion of population classified into the respective profiles at wave1/wave2/wave3.

To determine the numbers of profiles, two cohorts were analyzed. Summary of fit statistics for LPA for both samples are presented in S4 Table in supplements. All fit statistics for each time wave present a pattern of consistent decrease as the number of latent profiles goes up. Elbow plots then were drawn to help reducing the range of model selection (S3 Fig). A similar pattern was presented across time and sample. Elbow plots indicate the final solution is among 3 classes to 5 classes. LMR and BLRT suggest current models all fit well (S5 Table). Entropy values suggest classification qualities for these models are all good and very close ranging from 0.805 to 0.882. We therefore plotted the domain satisfaction profiles for these models across time (S4–S9 Figs). The 3-profile solution provided better model fit among the all 3 to 5-profile LPA solutions (S4 Table) at each wave. Profiles were labelled *Low*, *Moderate* and *High* subjective well-being, with *Low* comprising approximately 30% of the sample, *Moderate* 50% of the sample, and *High* approximately 20% of the sample. No profiles showed differentiations in subjective well-being domains over time (S10 Fig), and the additional profiles appear to be a split of existing profiles. For parsimony, the final solution for LPA was retained at 3-profile at each wave. LTA results further confirmed this solution (Fig 1, S11 Fig).

Transition probabilities were obtained from the final retained 3-profile LPA model for both cohorts (S6 Table). Table 2 presents three transition probability matrices for Cohort 2003, representing the movement among subjective well-being profiles. The matrix represents the transition probabilities between two consecutive waves and was computed based on the number of people at *n + 1* wave divided by number of people at wave *n*. Therefore, transition probabilities on the diagonal describe the proportions of population at wave *n* remaining in the same profile at wave *n + 1*, off-diagonal probabilities reflect movements to different profiles. Overall, across three waves, 44% to 78% of participants remained in the same profile. Stabilities for *Low* and *Moderate* subjective well-being profiles between two adjacent waves were higher than that from wave 1 to wave 3 suggesting longer-term stabilities decrease for these two profiles. *High* subjective well-being profile was less predictable compared to the two other profiles. We further explored individual transitions and movements across three waves using LTA models.

**Table 2:** Transition Probability for Cohort 2003.

***Latent Profile Analysis***			
		Wave1	
Wave2	L	M	H
L	**51%**	16%	5%
M	41%	**62%**	34%
H	7%	22%	**61%**
		Wave2	
Wave3	L	M	H
L	**63%**	12%	7%
M	33%	**70%**	20%
H	5%	18%	**73%**
		Wave1	
Wave3	L	M	H
L	**44%**	15%	6%
M	49%	**60%**	16%
H	7%	25%	**78%**
***Latent Transition Analysis***			
		Wave1	
Wave2→Wave3	L	M	H
L→L	**51.8%**	10.8%	2.3%
L→M	6.4%	7.8%	4.5%
L→H	0.8%	1.1%	0.7%
M→L	8.2%	7.1%	3.8%
M→M	25.2%	**50.8%**	36.1%
M→H	1.9%	4.8%	4.8%
H→L	1.2%	1.8%	2.3%
H→M	3.2%	8.9%	7.9%
H→H	1.4%	7.0%	**37.6%**

*Note*: L = low satisfaction profiles; M = moderate satisfaction profiles;

H = high satisfaction profiles.

#### LTA results

Longer term transition probabilities were investigated based on the subjective well-being statuses at first wave (Table 2). Stability was not as high as we expected, for *Low* (52%), *Moderate* (51%) and *High* (38%). The longer term transition probabilities parallel findings from the cross-sectional LPA. Compared to the individuals who began in the *high* subjective well-being group, individuals who began in the *Low* and *Moderate* groups were more likely to remain in the same status over time. Even for individuals who began in *Low* and *High* groups, the stage-sequential transition into the *Moderate* profile was most stable, with stability of 25.2% and 36.1% respectively.

**Fig 2:**
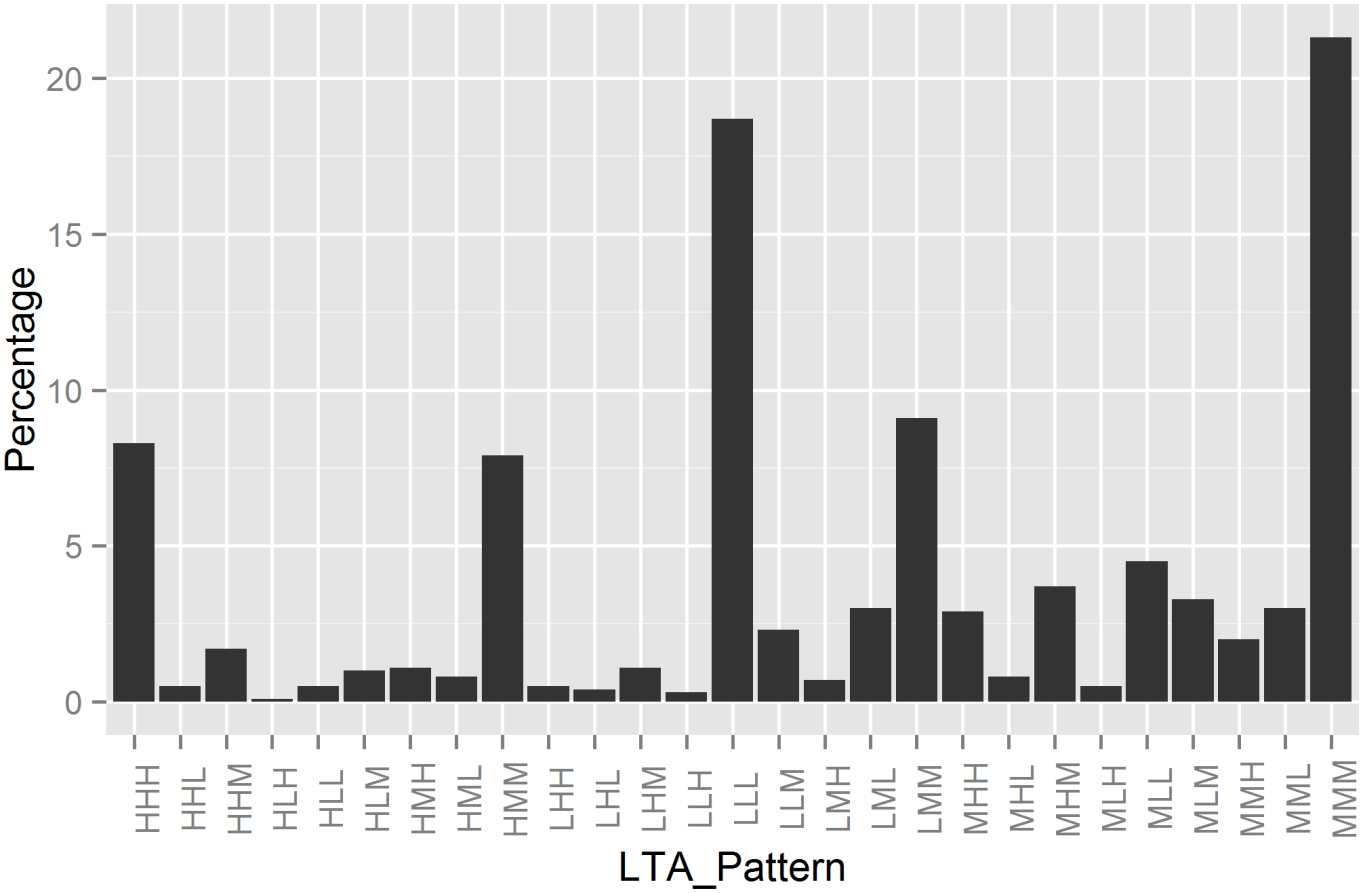
Percentage of population for each transition pattern based on three-wave latent transition analysis results for *Cohort 2003*. M = Moderate; L = Low; H = High. In this sense, LMH represents the transition pattern of L → M → H across three waves.

Fig 2 depicts the percentage of people for each transition pattern across three waves. Approximately half of participants (48%) remained in the same profile over time. 21% of participants remained in the *Moderate* profile (pattern MMM), 19% remained in the *Low* profile (pattern LLL), and 8% of participants remain in the *High* profile (pattern HHH). Second, the size of the pattern HHH is smaller than that of LMM (9.1%).

Despite the stable pattern (i.e., MMM, LLL, HHH), examinations of the transition direction revealed three main findings. First, Fig 2 shows two most prevalent transition patterns: one relates to individuals who move into a different profile then remain in it (e.g., LMM); the other relates to individuals who move into a different profile then move back to the same profile at baseline (e.g., LML). Individuals were also more likely to transition into these two patterns rather than transitioning into a different status on each wave (Table 2). Second, transitioning either one step up or one step down was most common, with transitions from H to L or vice versa least common. Third, no differences were found in terms of transitioning upward or downward, with participants equally likely to move up or move down over time (Fig 2, S7 Table).

### Determinants of Transition Patterns

Based on latent transition analysis results, three transition categories were defined: stable (48.3%), partially-stable (46.8%) and unstable (4.9%). Transition patterns having the same profile across three waves (e.g., MMM) were categorized as stable; patterns having two profiles the same were categorized as partially-stable (e.g., HHL or HLH); and patterns with different profiles on each wave (e.g., HLM) were categorized as unstable. A series of multinomial logistic regression analyses were conducted to compare the likelihood of belonging to unstable or partially-stable transition categories compared to stable transition category (the reference group).

#### Demographics

Gender had the most pronounced effect on transition pattern stability (χ^2^(2) = 9.74, *p* = 0.01), with a higher likelihood of males transiting into unstable profiles compared to stable profiles (Odds Ratio = 1.46, *p* = 0.00) (Table 3). There was no association between indigenous status and stability (χ^2^(2) = 0.23, *p* = 0.89). ‘Native’ and ‘First-Generation’ individuals, compared to ‘Non-native’ individuals, were 11% and 26% as likely to be in an unstable profile group rather than a stable group (Odds Ratio = 1.11, C.I. = (0.71, 1.73); Odds Ratio = 1.26, C.I. = (0.73, 2.17)). Overall, apart from strong gender effects, other demographic characteristics were not strongly associated with the long-term stability of subjective well-being.

**Table 3:** Influences of Demographics, Family, Life Path on Stability of Transition Patterns for cohort 2003.

		Unstable *vs*. Stable			Partially-Stable *vs*. Stable	
Effect	n	Odds Ratio (95%C.I.)	P-value	n	Odds Ratio (95%C.I.)	P-value
**Demographics**						
***Gender(N = 6204)***						
Male *vs*. Female	174	1.46(1.15, 1.85)	0.00	1420	1.01(0.92, 1.12)	0.79
***Indigenous(N = 6204)***						
Non-Indigenous *vs*. Indigenous	283	0.93(0.56, 1.53)	0.77	2739	0.95(0.76, 1.19)	0.66
***Immigration(N = 6110)***						
Native *vs*. Non-native	233	1.11(0.71, 1.73)	0.65	2316	0.99(0.82, 1.19)	0.91
First-Generation *vs*. Non-native	37	1.26(0.73, 2.17)	0.41	309	0.94(0.75, 1.19)	0.62
**Family**						
***SES(N = 6049)***						
White collar high skilled *vs*. Blue collar low skilled	196	0.79(0.51, 1.22)	0.29	1862	0.88(0.72, 1.08)	0.23
White collar low skilled *vs*. Blue collar low skilled	50	0.71(0.43, 1.18)	0.18	521	0.87(0.69, 1.09)	0.23
Blue collar high skilled *vs*. Blue collar low skilled	22	0.73(0.40, 1.34)	0.31	237	0.93(0.71, 1.22)	0.60
***Sibling(N = 6186)***						
No-Sibling *vs*. Having Sibling	11	0.55(0.29, 1.01)	0.06	179	0.94(0.76, 1.16)	0.57
***Family Structure(N = 6015)***						
Single Parent *vs*. Mixed	62	1.68(0.94, 3.01)	0.08	568	1.06(0.85, 1.31)	0.60
Nuclear *vs*. Mixed	218	1.78(1.04, 3.05)	0.04	2046	1.15(0.95, 1.39)	0.16
**Life Path**						
***Employment Wave1(N = 3774)***						
Employed *vs*. Unemployed	156	1.22(0.77, 1.95)	0.40	1503	1.01(0.84, 1.21)	0.93
***Employment Wave2(N = 3292)***						
Employed *vs*. Unemployed	142	0.68(0.36, 1.27)	0.23	1432	0.79(0.59, 1.06)	0.12
***Employment Wave3(N = 2399)***						
Employed *vs*. Unemployed	114	0.75(0.33, 1.68)	0.48	1063	0.94(0.63, 1.39)	0.75
***Education Wave1(N = 5202)***						
Studying *vs*. Not-studying	216	1.08(0.73, 1.58)	0.71	2109	1.11(0.94, 1.30)	0.22
***Education Wave2(N = 3616)***						
Studying *vs*. Not-studying	114	1.24(0.89, 1.72)	0.20	1004	0.93(0.81, 1.06)	0.27
***Education Wave3(N = 2642)***						
Studying *vs*. Not-studying	47	1.15(0.79, 1.67)	0.48	365	0.88(0.74, 1.05)	0.15

*Note*: Stable was selected as the reference category. C.I. = Confidence Interval; SES = Social Economic Status.

#### Family

Individuals from a higher SES family were more likely to remain in stable rather unstable or partially-stable profiles, although there was weak statistical evidence for this association (Table 3). Being from a family without a sibling had a substantially positive effect on long-term subjective well-being stability, with a no-sibling family less likely to have unstable (Odds Ratio = 0.55, C.I. = (0.29, 1.01) or partially-stable profiles (Odds Ratio = 0.94, C.I. = (0.76, 1.16)). In terms of the family structure, those individuals in single or nuclear families were more likely to be in unstable profiles compared to stable profiles (Odds Ratio = 1.68, C.I. = (0.94, 3.01); Odds Ratio = 1.78, C.I. = (1.04, 3.05)).

#### Life path

There was no association between employment and stability during post-high school transition. However employment apparently has mixed impact on individuals’ well-being before and after the post-high school transition. Before the transition, being employed increased the likelihood of being in unstable profiles (Odds Ratio = 1.22, C.I. = (0.77, 1.95)), while during the post-high school transition, employment substantially decreased the likelihood of being in an unstable (Odds Ratio = 0.68, C.I. = (0.36, 1.27); Odds Ratio = 0.75, C.I. = (0.36, 1.27)) compared to stable profiles. In contrast, at subsequent follow-up periods, those participants engaged in study were more likely to be in unstable or partially-stable profiles compared to those not engaged in study (Table 3).

## Discussion

This study assessed the long-term stability of subjective well-being during the post-school transition (i.e., transition from adolescence to young adulthood) in a population-based follow-up study of Australian young adults. First, this study identified 3 subjective well-being profiles, which confirmed that individuals have different levels of subjective well-being status. This finding is consistent with previous individual-level analyses findings that found three-level subgroups of subjective well-being [9,10]. However, for this age group from 17 to 25, subjective well-being status did not show substantial differentiations across life domains as was expected. It is possible that age group differences were amplified in previous studies when only average change patterns of life domains were compared.

Second, this study described individual transition behavior of subjective well-being status during a key transitional period from adolescence to young adulthood. Three transition patterns of subjective well-being were identified (stable, partially-stable, and unstable) representing particular underlying adaptation pathways during post-high school transition, and corroborated the basic principles of set-point theory [37]. According to set-point theory individuals have different but stable levels of subjective well-being. Following change in circumstances due to significant life events, for example a transition from high-school, set-point theory predicts a return to original subjective well-being status. Our findings revealed that most individuals remained in stable and in the ‘moderate’ subjective well-being status over time, and only a small proportion of people remained in high or low status over time. This is consistent with previous findings reporting positive levels of subjective well-being for the majority of youth [38]. This study also detected a large percentage of people who retained low levels of subject well-being (52%) or dropped to stable and low level of subjective well-being (13%) in the post-high school transition. Although these groups show stable or partially-stable patterns, they are also likely to be those groups vulnerable to the transition. This should be investigated in future studies.

Third, this study explored indicators associated with the stability of transition patterns. In particular, it was found that individuals who (a) were male, (b) from lower SES family, (c) from a family having siblings, (d) from single parent or nuclear family, (e) were unemployed, and (f) engaged in study during post-high school transition were more likely to have unstable transition patterns of subjective well-being status during post-high school transition. We found limited evidence of demographic variables in predicting stability. This may be due to the limited impact of demographic variables on youth subjective well-being [38]. The exception in the current study was for gender, where males were more likely to have unstable transition patterns. Second, the examination of the association between family indicators (Social Economic Status (SES), sibling, and family structure) and youth subjective well-being in previous studies produced controversial findings [38]. This study adds to the previous literatures by showing that there’s no association between family indicators and transition stability during post-high school transition. Third, this study demonstrated that unemployment and continued engagement in study after post-high school transition could lead to instability in subjective well-being. Benefits of employment to subjective well-being are obvious and have been well-documented [39]. The disadvantage of further involvement in study was not expected. The instability may be due to the delay of developmental tasks in other life domains, such as work, romantic relationships, and citizenship domains [40].

### Limitations

There are a number of methodological limitations that need to be considered in interpreting findings. First, the original subjective well-being scale was reduced to three major life domains (i.e., achievement, family, leisure). Although these three domains were confirmed by previous findings on this population [21,40], it is still possible this manipulation oversimplified relationships among life domains as individual’s weightings on life domains could vary [41]. Second, categorizing all the transition patterns into three stability status groups might oversimplify the heterogeneity of transition patterns. It is necessary for future research to directly examine the association between current indicators and subjective well-being change patterns. Third, although subjective well-being profiles identified here did not demonstrate substantial changes, it may be that subjective well-being change might emerge at later point in the life-course. For example, the cumulated stresses during transition may lead to the changes in subjective well-being in later adulthood. It also should be noted that the estimated individual’s latent profile membership may not directly correspond to their actual response [42]. That is, individuals in *Moderate* subjective well-being profiles may have high response on subjective well-being scale and vice versa. Finally, when analyzing the secondary data, it is inevitable some compromises need to be made between the complexity of the analysis and the relevance for the major research questions. One major compromise we made was the degree of the complexity of our *ad-hoc* analyses on latent transition analysis results. For example, the levels of effect of having-no-siblings on long-term subjective well-being stability have not been taken into account in this study, and the analysis on the directionality of transition patterns was not extensive enough. These are research questions left for future studies on the determinants of subjective well-being change patterns to explore. Likewise, a less sophisticated method for missing data (pairwise deletion) was employed for the multinomial logistic regression. More sophisticated missing data analysis is an avenue for future research.

### Implications

Findings here have important implications for both theory and practice. With respect to theory, identified transition patterns explained previous controversial findings relating to whether subjective well-being remains stable following significant life events. For most life events, previous studies observed no change or temporary change in subjective well-being status [43,44], while some life events show more enduring impact [45–47]. It is possible that previous research only captures one aspect of the transition patterns. For example, studies that observed no change or temporary change may identify the stable or partially-stable groups, but treated the proportionally small unstable groups as random errors. This study therefore demonstrated the efficiency of the individual-level analysis in the test of controversial findings. Second, further examinations of transition patterns could identify questions for future studies. For example, investigating stably-high groups could help answer questions relating to those characteristics that make people happy and less prone to poorer mental health. Likewise studying transition patterns of low subjective well-being groups could reveal precursors to negative affective outcomes such as depression. In practice, this study highlights the importance of incorporating person-focused perspectives in mental health intervention [48], that acknowledges population heterogeneity during periods of transition over the life course. In particular, by targeting those groups having stably-low or decreasing to low subjective well-being, mental health promotion and interventions could be applied more efficiently. Similarly, those unstable groups could inform the complexity and uncertainty of long-term subjective well-being change which might impact the outcome of mental health promotion and interventions. As post-school transition as well as the transition into young adulthood is the critical period for mental health promotion, the possible transition patterns and determinants for unstable transitions identified in this study is especially informative for those health professionals working with this population.

## Supporting Information

S1 Fig ESEM 3-factor solution. (DOCX)

S2 Fig Latent Profile Analyses for three domain satisfactions: achievement, family and leisure. (DOCX)

S3 Fig Elbow plots for Cohort 2003 and Cohort 1995 across 3 waves. (DOCX)

S4 Fig latent profile analysis solution for Cohort 2003 at wave 1. (DOCX)

S5 Fig latent profile analysis solution for Cohort 2003 at wave 2. (DOCX)

S6 Fig latent profile analysis solution for Cohort 2003 at wave 3. (DOCX)

S7 Fig latent profile analysis solution for Cohort 1995 at wave 1. (DOCX)

S8 Fig latent profile analysis solution for Cohort 1995 at wave 2. (DOCX)

S9 Fig latent profile analysis solution for Cohort 1995 at wave 3. (DOCX)

S10 Fig Subjective well-being profiles for two cohorts across time from cross-sectional latent profile analyses. Figure presented here is based on standardized scores.
(DOCX)

S11 Fig Subjective well-being profiles for two cohorts across time from three-wave latent transition analyses. Figure presented here is based on raw scores. Percentages represent the proportion of population classified into the respective profile at wave1/wave2/wave3.
(DOCX)

S1 Table Summary of model fit statistics for ESEM at 3 time waves. (DOCX)

S2 Table Factor loadings: 3-factor ESEM solutions based on responses to 11 items. (DOCX)

S3 Table Summary of model fit statistics for invariance test. (DOCX)

S4 Table Summary of model fit statistics for Latent Profile Analysis at 3 time waves. (DOCX)

S5 Table Summary of LMR and BLRT results for LPA. (DOCX)

S6 Table Transition Probability from Latent Profile Analysis. (DOCX)

S7 Table Demographics for each profile over time. (DOCX)

S1 Text Latent Profile Analysis. (DOCX)

S2 Text Measurement Invariance Test Results. (DOCX)
