# Supplementary figures and images for: Stability and Instability of Subjective Well-Being in the Transition from Adolescence to Young Adulthood: Longitudinal Evidence from 20991 Young Australians
Source: PLoS One. 2016 May 27;11(5):e0156399. doi: 10.1371/journal.pone.0156399 (PMC4883794; doi:10.1371/journal.pone.0156399)

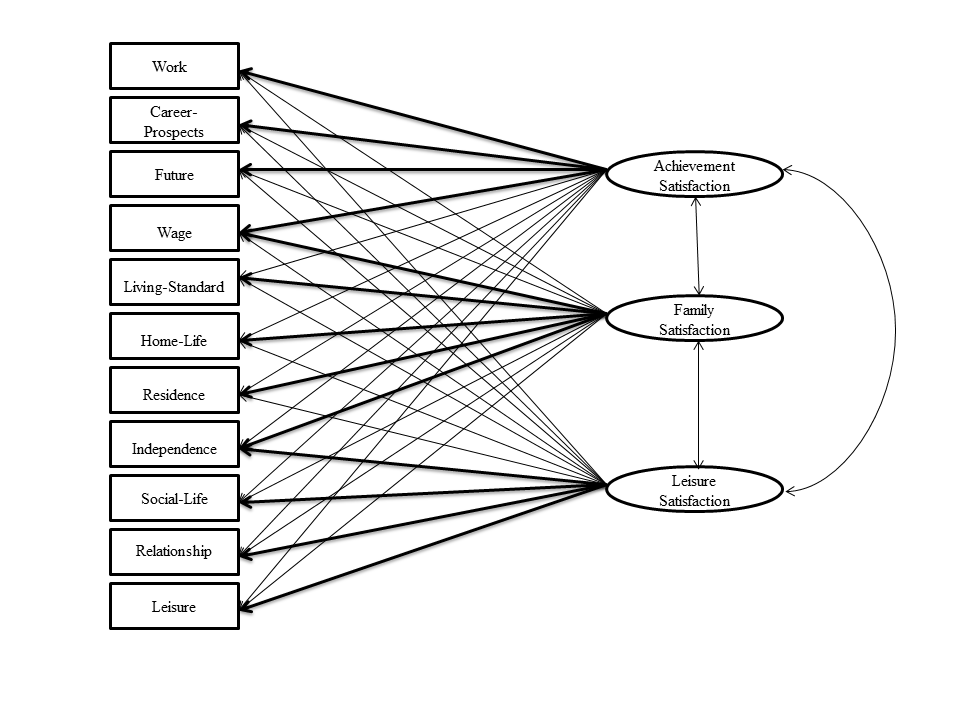


**S1 Fig. ESEM 3-factor solution.**

Supplement: S1 Fig — (DOCX) [file pone.0156399.s001.docx]

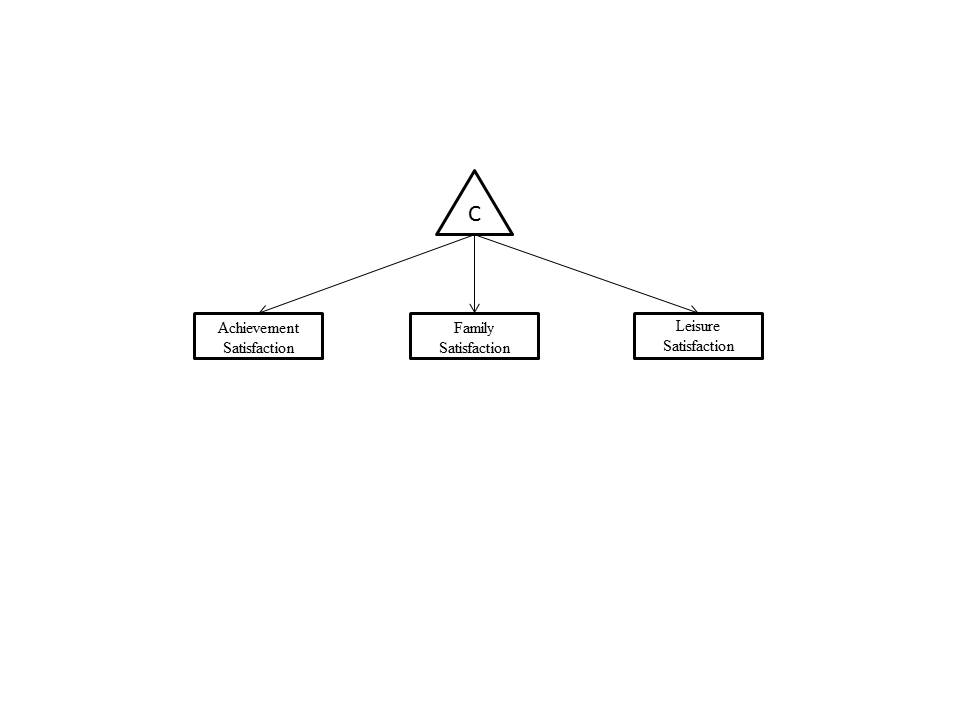


**S2 Fig. Latent Profile Analyses for three domain satisfactions: achievement, family and leisure.**

Supplement: S2 Fig — (DOCX) [file pone.0156399.s002.docx]

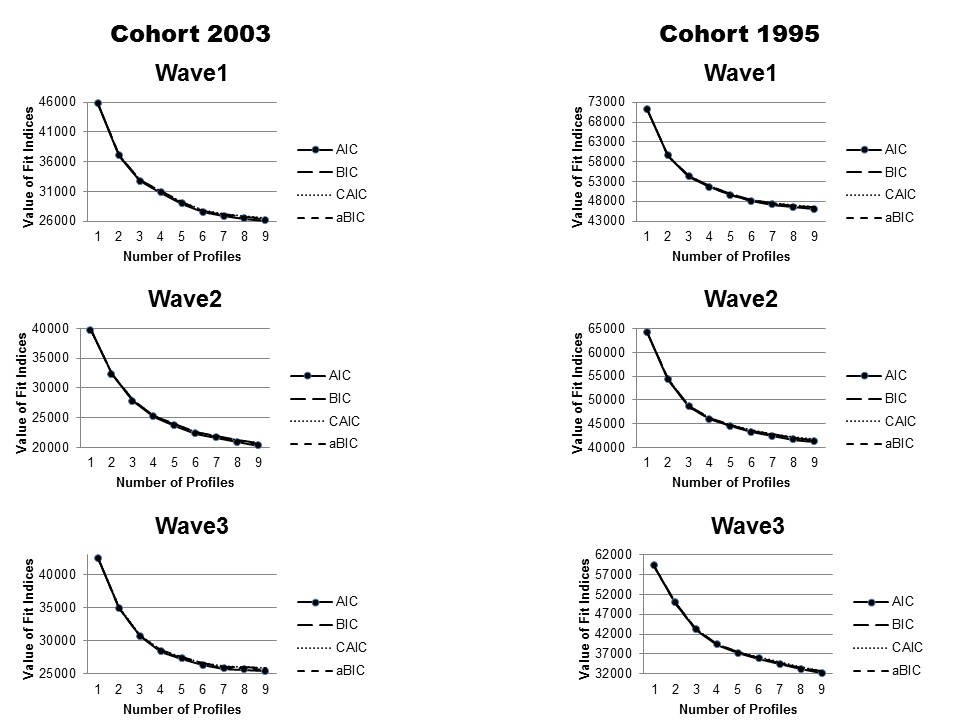


**S3 Fig. Elbow plots for Cohort 2003 and Cohort 1995 across 3 waves.**

Supplement: S3 Fig — (DOCX) [file pone.0156399.s003.docx]

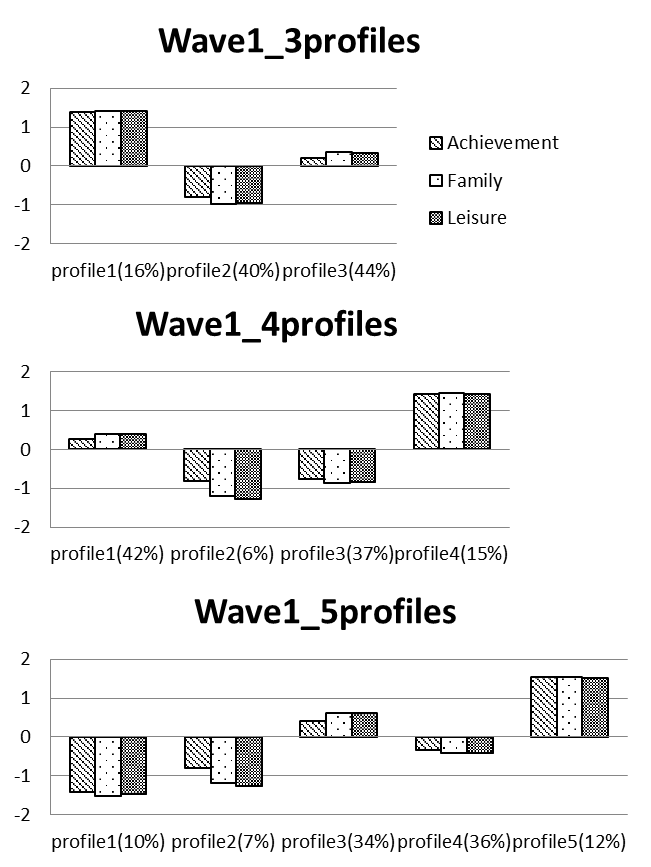


**S4 Fig. latent profile analysis solution for Cohort 2003 at wave 1.**

Supplement: S4 Fig — (DOCX) [file pone.0156399.s004.docx]

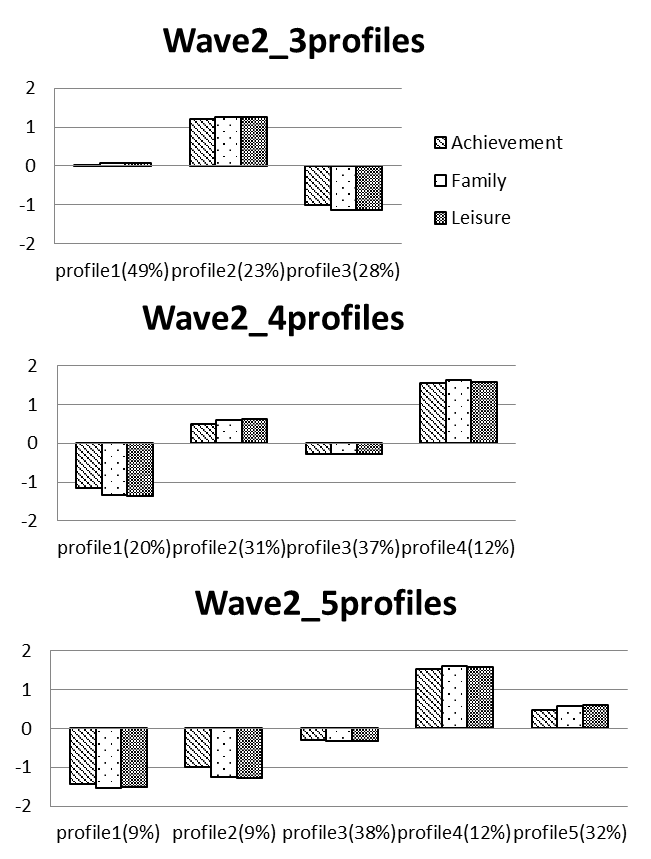


**S5 Fig. latent profile analysis solution for Cohort 2003 at wave 2.**

Supplement: S5 Fig — (DOCX) [file pone.0156399.s005.docx]

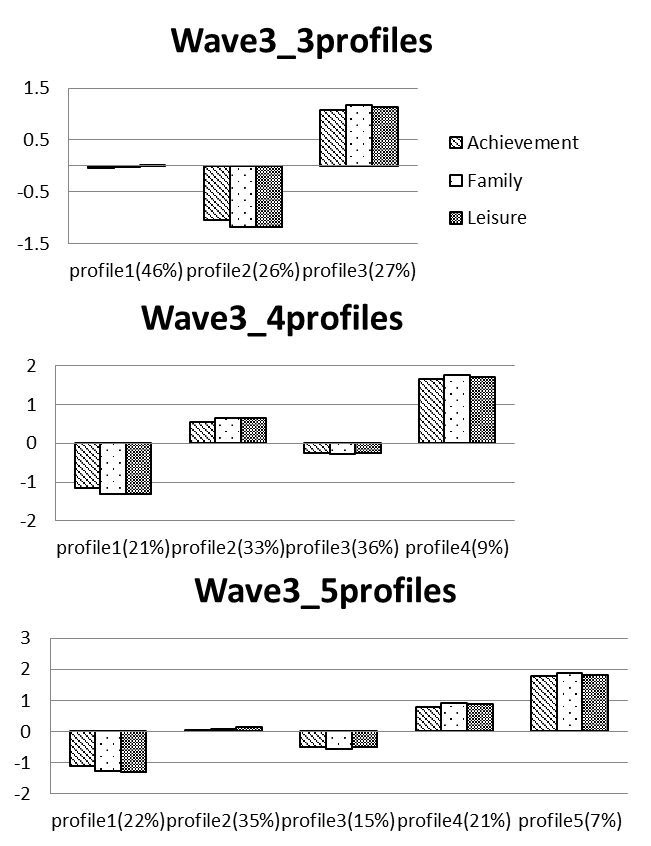


**S6 Fig. latent profile analysis solution for Cohort 2003 at wave 3.**

Supplement: S6 Fig — (DOCX) [file pone.0156399.s006.docx]

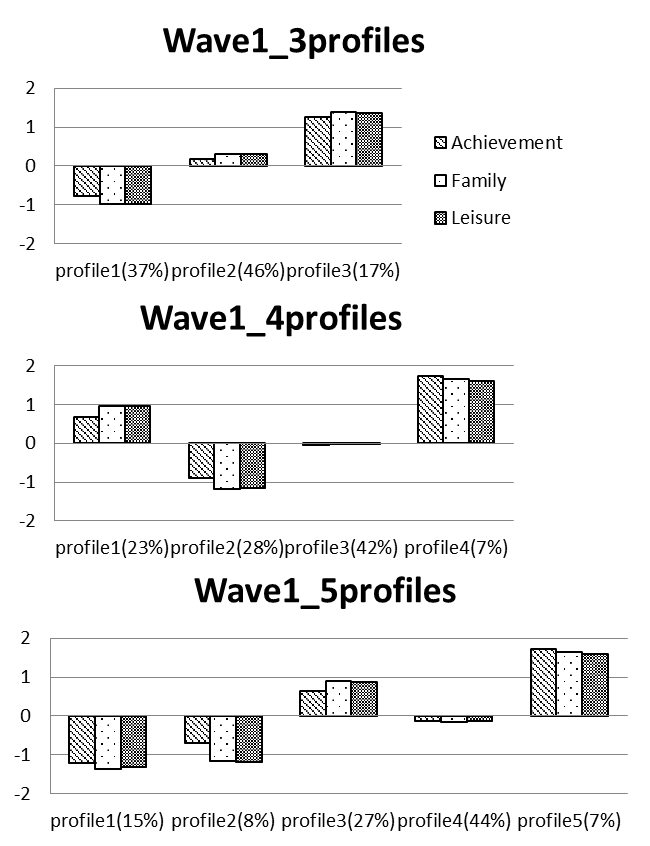


**S7 Fig. latent profile analysis solution for Cohort 1995 at wave 1.**

Supplement: S7 Fig — (DOCX) [file pone.0156399.s007.docx]

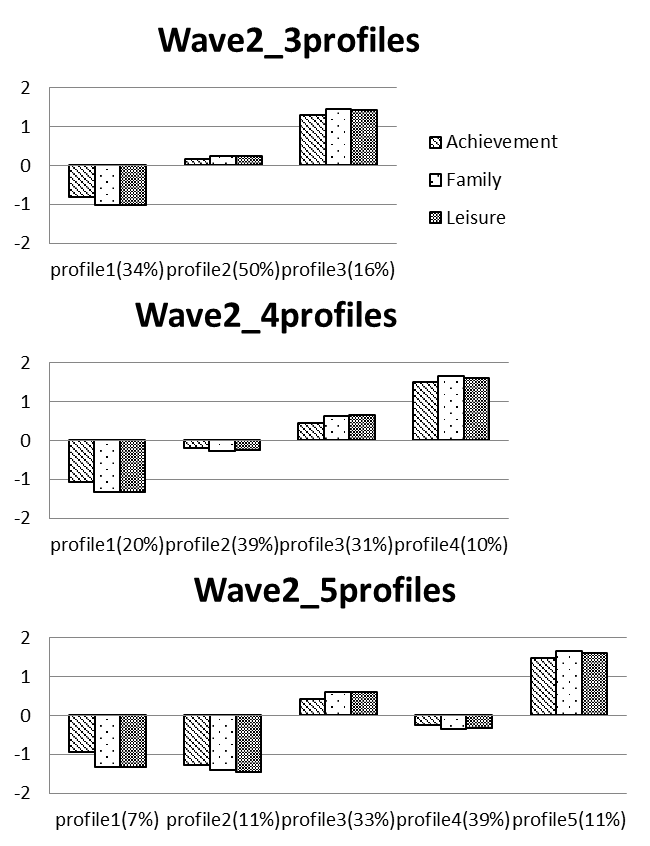


**S8 Fig. latent profile analysis solution for Cohort 1995 at wave 2.**

Supplement: S8 Fig — (DOCX) [file pone.0156399.s008.docx]

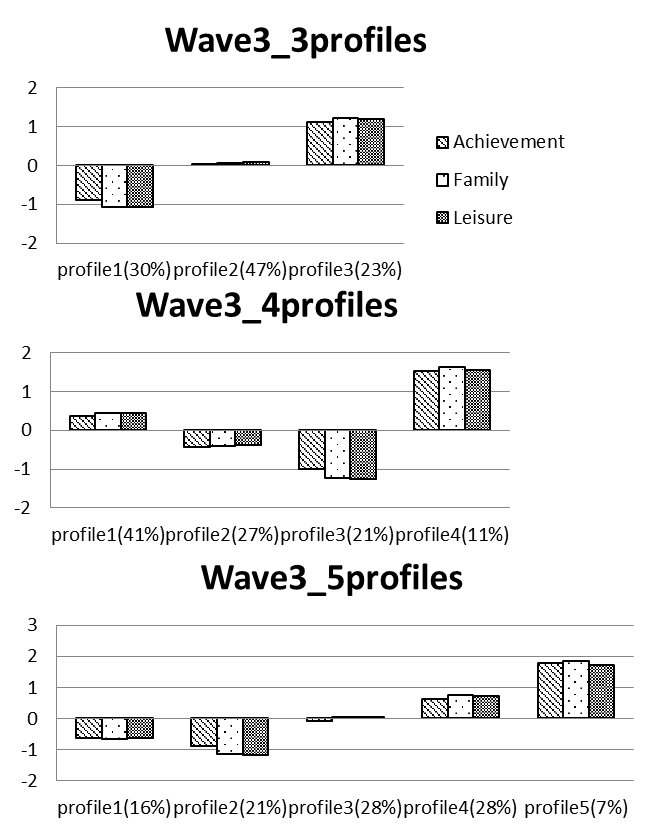


**S9 Fig. latent profile analysis solution for Cohort 1995 at wave 3.**

Supplement: S9 Fig — (DOCX) [file pone.0156399.s009.docx]
