# Supplementary material for: Stability and Instability of Subjective Well-Being in the Transition from Adolescence to Young Adulthood: Longitudinal Evidence from 20991 Young Australians
Source: PLoS One. 2016 May 27;11(5):e0156399. doi: 10.1371/journal.pone.0156399 (PMC4883794; doi:10.1371/journal.pone.0156399)
Supplement: S10 Fig — Figure presented here is based on standardized scores. (DOCX) [file pone.0156399.s010.docx]

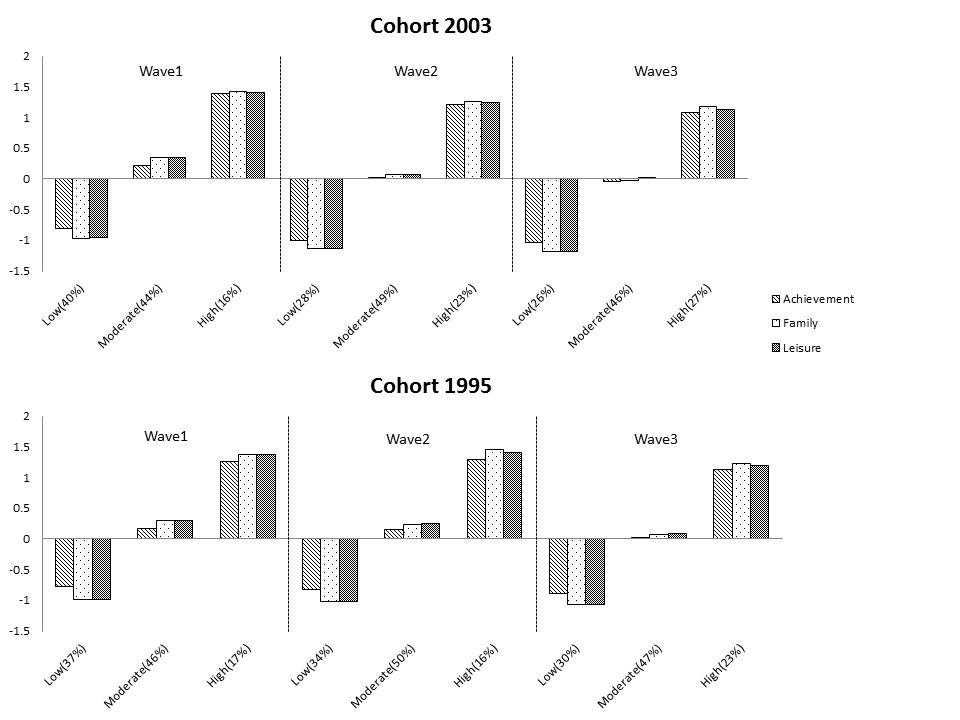


**S10 Fig. Subjective well-being profiles for two cohorts across time from cross-sectional latent profile analyses.** Figure presented here is based on standardized scores.
