# Supplementary material for: Stability and Instability of Subjective Well-Being in the Transition from Adolescence to Young Adulthood: Longitudinal Evidence from 20991 Young Australians
Source: PLoS One. 2016 May 27;11(5):e0156399. doi: 10.1371/journal.pone.0156399 (PMC4883794; doi:10.1371/journal.pone.0156399)
Supplement: S1 Table — (DOCX) [file pone.0156399.s012.docx]

**S1 Table***.* **Summary of model fit statistics for ESEM at 3 time waves.**

| **Model** | **χ^2^** | ***df*** | **CFI** | **TLI** | **RMSEA(90%CI)** |
| --- | --- | --- | --- | --- | --- |
| **Cohort 2003** | | | | | |
| ***Wave 1 (Grade 12)*** |  |  |  |  |  |
| 1 factor_ESEM | 2853.495* | 44 | 0.929 | 0.911 | 0.102(0.098, 0.105) |
| 2 factor_ESEM | 968.259* | 34 | 0.976 | 0.962 | 0.067(0.063, 0.070) |
| 3 factor_ESEM | 248.594* | 25 | 0.994 | 0.988 | 0.038(0.034, 0.042) |
| 4 factor_ESEM | 99.221* | 17 | 0.998 | 0.993 | 0.028(0.023, 0.033) |
| ***Wave 2*** |  |  |  |  |  |
| 1 factor_ESEM | 2202.794* | 44 | 0.935 | 0.919 | 0.106(0.103, 0.110) |
| 2 factor_ESEM | 970.635* | 34 | 0.972 | 0.954 | 0.080(0.075, 0.084) |
| 3 factor_ESEM | 256.531* | 25 | 0.993 | 0.985 | 0.046(0.041, 0.051) |
| 4 factor_ESEM | 120.711* | 17 | 0.997 | 0.990 | 0.038(0.031, 0.044) |
| ***Wave 3*** |  |  |  |  |  |
| 1 factor_ESEM | 1882.273* | 44 | 0.939 | 0.924 | 0.115(0.111, 0.120) |
| 2 factor_ESEM | 628.750* | 34 | 0.980 | 0.968 | 0.074(0.069, 0.080) |
| 3 factor_ESEM | 203.542* | 25 | 0.994 | 0.987 | 0.048(0.042, 0.054) |
| 4 factor_ESEM | NA | NA | NA | NA | NA |
|  |  |  |  |  |  |
| **Cohort 1995** | | | | | |
| ***Wave 1 (Grade 12)*** |  |  |  |  |  |
| 1 factor_ESEM | 5342.665* | 44 | 0.905 | 0.881 | 0.111(0.109, 0.114) |
| 2 factor_ESEM | 1550.101* | 34 | 0.973 | 0.956 | 0.068(0.065, 0.071) |
| 3 factor_ESEM | 281.464* | 25 | 0.995 | 0.990 | 0.032(0.029, 0.036) |
| 4 factor_ESEM | 109.983* | 17 | 0.998 | 0.995 | 0.024(0.020, 0.028) |
| ***Wave 2*** |  |  |  |  |  |
| 1 factor_ESEM | 3429.670* | 44 | 0.920 | 0.900 | 0.106(0.103, 0.109) |
| 2 factor_ESEM | 1245.745* | 34 | 0.971 | 0.954 | 0.072(0.069, 0.075) |
| 3 factor_ESEM | 216.321* | 25 | 0.995 | 0.990 | 0.033(0.029, 0.038) |
| 4 factor_ESEM | NA | NA | NA | NA | NA |
| ***Wave 3*** |  |  |  |  |  |
| 1 factor_ESEM | 2472.848* | 44 | 0.913 | 0.891 | 0.109(0.105, 0.113) |
| 2 factor_ESEM | 881.206* | 34 | 0.970 | 0.951 | 0.073(0.069, 0.077) |
| 3 factor_ESEM | 295.254* | 25 | 0.990 | 0.979 | 0.048(0.043, 0.053) |
| 4 factor_ESEM | NA | NA | NA | NA | NA |

*Note*: **p* < .01; **χ** ^2^: Robust weighted least square chi-square; *df*: Degree of freedom; CFI: Comparative fit index; TLI: Tucker-Lewis index; RMSEA: Root mean square error of approximation; RMSEA 90% CI: 90% Confidence interval for the RMSEA point estimate.
