# Supplementary material for: Stability and Instability of Subjective Well-Being in the Transition from Adolescence to Young Adulthood: Longitudinal Evidence from 20991 Young Australians
Source: PLoS One. 2016 May 27;11(5):e0156399. doi: 10.1371/journal.pone.0156399 (PMC4883794; doi:10.1371/journal.pone.0156399)
Supplement: S2 Table — (DOCX) [file pone.0156399.s013.docx]

**S2 Table.** **Factor loadings: 3-factor ESEM solutions based on responses to 11 items.**

| **Items** |  | **Cohort2003** |  |  |  | **Cohort1995** |  |
| --- | --- | --- | --- | --- | --- | --- | --- |
|  | **Wave1** | **Wave2** | **Wave3** |  | **Wave1** | **Wave2** | **Wave3** |
| ***Leisure Satisfaction*** |  |  |  |  |  |  |  |
| Work | 0.137 | 0.161 | 0.115 |  | 0.102 | 0.096 | 0.049 |
| Leisure | **0.512** | **0.601** | **0.606** |  | **0.572** | **0.597** | **0.647** |
| Relationship | **0.534** | **0.493** | **0.576** |  | **0.438** | **0.426** | **0.405** |
| Wages | 0.053 | -0.029 | -0.059 |  | 0.057 | 0.002 | 0.057 |
| Social-Life | **0.824** | **0.821** | **0.751** |  | **0.859** | **0.874** | **0.847** |
| Independence | **0.339** | **0.342** | **0.390** |  | **0.372** | **0.309** | **0.296** |
| Career-Prospects | -0.007 | 0.049 | 0.064 |  | 0.024 | 0.040 | 0.061 |
| Future | 0.149 | 0.143 | 0.169 |  | 0.089 | 0.141 | 0.195 |
| Home-Life | 0.185 | 0.236 | 0.273 |  | 0.146 | 0.162 | 0.166 |
| Living-Standard | 0.146 | 0.102 | 0.115 |  | 0.143 | 0.130 | 0.146 |
| Residence | 0.088 | 0.104 | 0.117 |  | 0.109 | 0.098 | 0.136 |
| ***Achievement Satisfaction*** |  |  |  |  |  |  |  |
| Work | **0.452** | **0.484** | **0.526** |  | **0.399** | **0.480** | **0.547** |
| Leisure | 0.172 | 0.126 | 0.117 |  | 0.096 | 0.109 | 0.071 |
| Relationship | 0.157 | 0.225 | 0.170 |  | 0.175 | 0.151 | 0.180 |
| Wages | **0.274** | **0.283** | **0.378** |  | **0.240** | **0.301** | **0.359** |
| Social-Life | -0.012 | 0.038 | 0.047 |  | 0.020 | 0.034 | 0.043 |
| Independence | 0.163 | 0.162 | 0.170 |  | 0.110 | 0.111 | 0.151 |
| Career-Prospects | **0.835** | **0.867** | **0.856** |  | **0.852** | **0.870** | **0.884** |
| Future | **0.717** | **0.641** | **0.701** |  | **0.752** | **0.645** | **0.586** |
| Home-Life | 0.103 | 0.109 | 0.106 |  | 0.118 | 0.102 | 0.051 |
| Living-Standard | 0.106 | 0.135 | 0.138 |  | 0.119 | 0.119 | 0.203 |
| Residence | 0.059 | 0.011 | 0.002 |  | 0.016 | 0.020 | -0.015 |
| ***Family Satisfaction*** |  |  |  |  |  |  |  |
| Work | 0.193 | 0.134 | 0.186 |  | 0.264 | 0.188 | 0.177 |
| Leisure | 0.153 | 0.129 | 0.160 |  | 0.150 | 0.119 | 0.125 |
| Relationship | 0.152 | 0.167 | 0.132 |  | 0.234 | 0.272 | 0.280 |
| Wages | **0.261** | **0.349** | **0.336** |  | **0.317** | **0.257** | **0.245** |
| Social-Life | 0.056 | 0.030 | 0.085 |  | -0.014 | -0.006 | 0.013 |
| Independence | **0.246** | **0.326** | **0.319** |  | **0.252** | **0.355** | **0.392** |
| Career-Prospects | 0.042 | -0.034 | 0.004 |  | -0.020 | -0.054 | -0.067 |
| Future | 0.065 | 0.169 | 0.113 |  | 0.067 | 0.132 | 0.189 |
| Home-Life | **0.640** | **0.610** | **0.588** |  | **0.655** | **0.645** | **0.687** |
| Living-Standard | **0.687** | **0.643** | **0.659** |  | **0.643** | **0.618** | **0.559** |
| Residence | **0.699** | **0.732** | **0.689** |  | **0.683** | **0.691** | **0.656** |

*Note:* the major loading is bolded.
