# Supplementary material for: Stability and Instability of Subjective Well-Being in the Transition from Adolescence to Young Adulthood: Longitudinal Evidence from 20991 Young Australians
Source: PLoS One. 2016 May 27;11(5):e0156399. doi: 10.1371/journal.pone.0156399 (PMC4883794; doi:10.1371/journal.pone.0156399)
Supplement: S3 Table — (DOCX) [file pone.0156399.s014.docx]

**S3 Table.** **Summary of model fit statistics for invariance test.**

| **Model** | **χ** **^2^(*df*)** | **CFI** | **TLI** | **RMSEA(90%CI)** | **MDΔχ²(*df*)** | **ΔCFI** | **ΔRMSEA** |
| --- | --- | --- | --- | --- | --- | --- | --- |
| **Cohort 2003** | | | | | | | |
| Configural Invariance | 970.391*(378) | 0.994 | 0.991 | 0.016(0.015, 0.017) | NA | NA | NA |
| Weak Invariance | 1026.624*(426) | 0.994 | 0.992 | 0.015(0.014, 0.016) | 83.428*(48) | 0.000 | -0.001 |
| Strong Invariance | 1396.514*(486) | 0.990 | 0.989 | 0.017(0.016, 0.018) | 371.842*(60) | -0.004 | 0.002 |
| Strict Invariance | 1748.875*(508) | 0.987 | 0.986 | 0.020(0.019, 0.021) | 286.453*(22) | -0.003 | 0.003 |
| Variance-Covariance Invariance | 1883.190*(520) | 0.985 | 0.985 | 0.021(0.020, 0.022) | 109.513*(12) | -0.002 | 0.001 |
| Latent Mean Invariance | 2181.407*(526) | 0.982 | 0.982 | 0.023(0.022, 0.024) | 158.352*(6) | -0.003 | 0.002 |
| **Cohort 1995** | | | | | | | |
| Configural Invariance | 976.316*(378) | 0.993 | 0.991 | 0.010(0.010, 0.011) | NA | NA | NA |
| Weak Invariance | 1205.014*(426) | 0.993 | 0.991 | 0.010(0.010, 0.011) | 555.509*(144) | -0.001 | 0.001 |
| Strong Invariance | 1997.181*(486) | 0.991 | 0.990 | 0.011(0.011, 0.011) | 1767.182*(180) | -0.006 | 0.004 |
| Strict Invariance | 2492.107*(508) | 0.990 | 0.989 | 0.012(0.011, 0.012) | 1087.695*(66) | -0.004 | 0.002 |
| Variance-Covariance Invariance | 2551.986*(520) | 0.989 | 0.988 | 0.012(0.012, 0.012) | 591.630*(36) | -0.001 | 0.000 |
| Latent Mean Invariance | 2914.661*(526) | 0.987 | 0.986 | 0.013(0.013, 0.013) | 796.767*(18) | -0.003 | 0.002 |

*Note*: **p* < .01; **χ** ^2^: Robust weighted least square chi-square; *df*: Degree of freedom; CFI: Comparative fit index; TLI: Tucker-Lewis index; RMSEA: Root mean square error of approximation; RMSEA 90% CI: 90% Confidence interval for the RMSEA point estimate;

MD∆χ^2^: Change in χ^2^ relative to the preceding model calculated from Mplus DIFFTEST function; ∆CFI: Change in comparative fit index relative to the preceding model; ∆RMSEA: Change in root mean square error of approximation relative to the preceding model.
