# Supplementary material for: Stability and Instability of Subjective Well-Being in the Transition from Adolescence to Young Adulthood: Longitudinal Evidence from 20991 Young Australians
Source: PLoS One. 2016 May 27;11(5):e0156399. doi: 10.1371/journal.pone.0156399 (PMC4883794; doi:10.1371/journal.pone.0156399)
Supplement: S4 Table — (DOCX) [file pone.0156399.s015.docx]

| **S4 Table. Summary of model fit statistics for Latent Profile Analysis at 3 time waves.** | | | | | | | |
| --- | --- | --- | --- | --- | --- | --- | --- |
|  | | | | | | |  |
| **Model** | **LL** | **# Parameters** | **AIC** | **CAIC** | **BIC** | **ABIC** | **Entropy** |
| **Cohort 2003** | | | | | | | |
| ***Wave 1 (Grade12)*** | |  |  |  |  |  |  |
| One profile | -22864.3 | 6 | 45740.64 | 45740.64 | 45781.03 | 45761.97 | NA |
| Two profiles | -18483 | 13 | 36992.01 | 36992.01 | 37079.54 | 37038.23 | 0.837 |
| **Three profiles** | **-16328.6** | **20** | **32697.25** | **32697.25** | **32831.91** | **32768.35** | **0.876** |
| **Four profiles** | **-15371.5** | **27** | **30797.07** | **30797.07** | **30978.85** | **30893.06** | **0.882** |
| **Five profiles** | **-14424.1** | **34** | **28916.27** | **28916.27** | **29145.19** | **29037.15** | **0.875** |
| Six profiles | -13696.3 | 41 | 27474.66 | 27474.66 | 27750.71 | 27620.42 | 0.879 |
| Seven profiles | -13368 | 48 | 26832.07 | 26832.07 | 27155.25 | 27002.72 | 0.851 |
| Eight profiles | -13134.3 | 55 | 26378.69 | 26378.69 | 26749 | 26574.22 | 0.861 |
| Nine profiles | -12968.8 | 62 | 26061.53 | 26061.53 | 26478.97 | 26281.96 | 0.84 |
| ***Wave 2*** |  |  |  |  |  |  |  |
| One profile | -21241 | 6 | 42494 | 42494 | 42534.4 | 42515.33 | NA |
| Two profiles | -17449.3 | 13 | 34924.58 | 34924.58 | 35012.11 | 34970.8 | 0.803 |
| **Three profiles** | **-15300** | **20** | **30640.05** | **30640.05** | **30774.71** | **30711.16** | **0.833** |
| **Four profiles** | **-14144.3** | **27** | **28342.57** | **28342.57** | **28524.36** | **28438.57** | **0.848** |
| **Five profiles** | **-13588.8** | **34** | **27245.58** | **27245.58** | **27474.5** | **27366.46** | **0.86** |
| Six profiles | -13105.7 | 41 | 26293.34 | 26293.34 | 26569.39 | 26439.1 | 0.837 |
| Seven profiles | -12825.8 | 48 | 25747.62 | 25747.62 | 26070.8 | 25918.27 | 0.838 |
| Eight profiles | -12752.5 | 55 | 25615 | 25615 | 25985.31 | 25810.53 | 0.849 |
| Nine profiles | -12620.6 | 62 | 25365.25 | 25365.25 | 25782.69 | 25585.67 | 0.848 |
| ***Wave 3*** |  |  |  |  |  |  |  |
| One profile | -19847.4 | 6 | 39706.76 | 39706.76 | 39747.16 | 39728.1 | NA |
| Two profiles | -16131.5 | 13 | 32289.09 | 32289.09 | 32376.61 | 32335.3 | 0.791 |
| **Three profiles** | **-13868.3** | **20** | **27776.66** | **27776.66** | **27911.32** | **27847.76** | **0.828** |
| **Four profiles** | **-12533.5** | **27** | **25120.99** | **25120.99** | **25302.78** | **25216.98** | **0.861** |
| **Five profiles** | **-11789.8** | **34** | **23647.69** | **23647.69** | **23876.61** | **23768.57** | **0.863** |
| Six profiles | -11119.9 | 41 | 22321.88 | 22321.88 | 22597.93 | 22467.64 | 0.852 |
| Seven profiles | -10762.1 | 48 | 21620.14 | 21620.14 | 21943.32 | 21790.79 | 0.852 |
| Eight profiles | -10360.8 | 55 | 20831.58 | 20831.58 | 21201.89 | 21027.12 | 0.825 |
| Nine profiles | -10091.8 | 62 | 20307.52 | 20307.52 | 20724.96 | 20527.94 | 0.831 |
| **Cohort 1995** | | | | | | | |
| ***Wave 1*** |  |  |  |  |  |  |  |
| One profile | -35628.1 | 6 | 71268.19 | 71268.19 | 71311.29 | 71292.23 | NA |
| Two profiles | -29700.3 | 13 | 59426.51 | 59426.51 | 59519.89 | 59478.58 | 0.819 |
| **Three profiles** | **-27094.7** | **20** | **54229.33** | **54229.33** | **54373.01** | **54309.45** | **0.844** |
| **Four profiles** | **-25751.5** | **27** | **51556.91** | **51556.91** | **51750.87** | **51665.07** | **0.845** |
| **Five profiles** | **-24705** | **34** | **49477.98** | **49477.98** | **49722.23** | **49614.18** | **0.861** |
| Six profiles | -23940.3 | 41 | 47962.62 | 47962.62 | 48257.16 | 48126.87 | 0.858 |
| Seven profiles | -23510.9 | 48 | 47117.87 | 47117.87 | 47462.69 | 47310.15 | 0.835 |
| Eight profiles | -23209.8 | 55 | 46529.59 | 46529.59 | 46924.7 | 46749.92 | 0.793 |
| Nine profiles | -22926.8 | 62 | 45977.62 | 45977.62 | 46423.01 | 46225.99 | 0.786 |
| ***Wave 2*** |  |  |  |  |  |  |  |
| One profile | -32091.6 | 6 | 64195.15 | 64195.15 | 64238.25 | 64219.19 | NA |
| Two profiles | -27124.7 | 13 | 54275.43 | 54275.43 | 54368.82 | 54327.5 | 0.76 |
| **Three profiles** | **-24237.5** | **20** | **48515.06** | **48515.06** | **48658.74** | **48595.18** | **0.831** |
| **Four profiles** | **-22916.1** | **27** | **45886.2** | **45886.2** | **46080.16** | **45994.36** | **0.819** |
| **Five profiles** | **-22197.1** | **34** | **44462.19** | **44462.19** | **44706.43** | **44598.39** | **0.836** |
| Six profiles | -21576.3 | 41 | 43234.6 | 43234.6 | 43529.13 | 43398.84 | 0.821 |
| Seven profiles | -21136 | 48 | 42368.03 | 42368.03 | 42712.85 | 42560.31 | 0.823 |
| Eight profiles | -20770.9 | 55 | 41651.79 | 41651.79 | 42046.9 | 41872.12 | 0.831 |
| Nine profiles | -20534.3 | 62 | 41192.64 | 41192.64 | 41638.03 | 41441 | 0.787 |
| ***Wave 3*** |  |  |  |  |  |  |  |
| One profile | -29598.1 | 6 | 59208.14 | 59208.14 | 59251.24 | 59232.18 | NA |
| Two profiles | -24940.6 | 13 | 49907.21 | 49907.21 | 50000.6 | 49959.29 | 0.739 |
| **Three profiles** | **-21505.4** | **20** | **43050.78** | **43050.78** | **43194.46** | **43130.9** | **0.805** |
| **Four profiles** | **-19632.2** | **27** | **39318.34** | **39318.34** | **39512.3** | **39426.5** | **0.833** |
| **Five profiles** | **-18573.3** | **34** | **37214.68** | **37214.68** | **37458.93** | **37350.88** | **0.822** |
| Six profiles | -17843.6 | 41 | 35769.2 | 35769.2 | 36063.74 | 35933.45 | 0.824 |
| Seven profiles | -17165.2 | 48 | 34426.35 | 34426.35 | 34771.17 | 34618.63 | 0.797 |
| Eight profiles | -16502.2 | 55 | 33114.33 | 33114.33 | 33509.43 | 33334.65 | 0.807 |
| Nine profiles | -16011.6 | 62 | 32147.15 | 32147.15 | 32592.54 | 32395.51 | 0.816 |
| *Note*: LL: Log Likelihood; AIC: Akaike Information Criterion; BIC: the Bayesian Information Criterion; | | | | | | | |
| CAIC: the Consistent Akaike Information Cariterion; ABIC: Adjusted BIC. | | | | | | |  |
