# Supplementary material for: Stability and Instability of Subjective Well-Being in the Transition from Adolescence to Young Adulthood: Longitudinal Evidence from 20991 Young Australians
Source: PLoS One. 2016 May 27;11(5):e0156399. doi: 10.1371/journal.pone.0156399 (PMC4883794; doi:10.1371/journal.pone.0156399)
Supplement: S5 Table — (DOCX) [file pone.0156399.s016.docx]

| **S5 Table.** **Summary of LMR and BLRT results for LPA** | | |
| --- | --- | --- |
| **Model** | **LMR(*p*)** | **BLRT(*p*)** |
| **Cohort2003** |  |  |
| ***Wave1*** |  |  |
| 3 classes | 0 | 0 |
| 4 classes | 0 | 0 |
| 5 classes | 0 | 0 |
| ***Wave2*** |  |  |
| 3 classes | 0 | 0 |
| 4 classes | 0 | 0 |
| 5 classes | 0 | 0 |
| ***Wave3*** |  |  |
| 3 classes | 0 | 0 |
| 4 classes | 0 | 0 |
| 5 classes | 0 | 0 |
| **Cohort1995** |  |  |
| ***Time1*** |  |  |
| 3 classes | 0 | 0 |
| 4 classes | 0 | 0 |
| 5 classes | 0 | 0 |
| ***Time2*** |  |  |
| 3 classes | 0 | 0 |
| 4 classes | 0 | 0 |
| 5 classes | 0 | 0 |
| ***Time3*** |  |  |
| 3 classes | 0 | 0 |
| 4 classes | 0 | 0 |
| 5 classes | 0.0024 | 0 |
| *Note*. LMR: Lo, Mendel, & Rubin likelihood | | |
| ratio test; BLRT: Bootstrap Likelihood Ratio Test. | | |
