# Supplementary material for: Stability and Instability of Subjective Well-Being in the Transition from Adolescence to Young Adulthood: Longitudinal Evidence from 20991 Young Australians
Source: PLoS One. 2016 May 27;11(5):e0156399. doi: 10.1371/journal.pone.0156399 (PMC4883794; doi:10.1371/journal.pone.0156399)
Supplement: S6 Table — (DOCX) [file pone.0156399.s017.docx]

**S6 Table.** **Transition Probability from Latent Profile Analysis.**

|  | **Cohort 2003** | |  |  |  | **Cohort 1995** | |  |
| --- | --- | --- | --- | --- | --- | --- | --- | --- |
|  |  | Wave1 |  |  |  |  | Wave1 |  |
| Wave2 | L | M | H |  | Wave2 | L | M | H |
| L | **51%** | 16% | 5% |  | L | **62%** | 19% | 9% |
| M | 41% | **62%** | 34% |  | M | 33% | **67%** | 53% |
| H | 7% | 22% | **61%** |  | H | 5% | 14% | **38%** |
|  |  | Wave2 |  |  |  |  | Wave2 |  |
| Wave3 | L | M | H |  | Wave3 | L | M | H |
| L | **63%** | 12% | 7% |  | L | **68%** | 11% | 6% |
| M | 33% | **70%** | 20% |  | M | 25% | **70%** | 24% |
| H | 5% | 18% | **73%** |  | H | 7% | 20% | **71%** |
|  |  | Wave1 |  |  |  |  | Wave1 |  |
| Wave3 | L | M | H |  | Wave3 | L | M | H |
| L | **44%** | 15% | 6% |  | L | **56%** | 16% | 6% |
| M | 49% | **60%** | 16% |  | M | 37% | **63%** | 31% |
| H | 7% | 25% | **78%** |  | H | 8% | 20% | **63%** |

Note: L = low satisfaction profiles; M = moderate satisfaction profiles;

H = high satisfaction profiles.
