# Supplementary material for: Stability and Instability of Subjective Well-Being in the Transition from Adolescence to Young Adulthood: Longitudinal Evidence from 20991 Young Australians
Source: PLoS One. 2016 May 27;11(5):e0156399. doi: 10.1371/journal.pone.0156399 (PMC4883794; doi:10.1371/journal.pone.0156399)
Supplement: S7 Table — (DOCX) [file pone.0156399.s018.docx]

**S7 Table*.* Demographics for each profile over time.**

|  | High | Low | Moderate |
| --- | --- | --- | --- |
| **Gender** |  |  |  |
| *Male* |  |  |  |
| Wave1 | 638(21%) | 1098(37%) | 1247(42%) |
| Wave2 | 587(20%) | 915(31%) | 1481(50%) |
| Wave3 | 470(16%) | 941(32%) | 1572(53%) |
| *Female* |  |  |  |
| Wave1 | 703(22%) | 1102(35%) | 1322(42%) |
| Wave2 | 632(20%) | 985(31%) | 1510(48%) |
| Wave3 | 524(17%) | 1017(33%) | 1586(51%) |
| **Immigration** |  |  |  |
| *Native* |  |  |  |
| Wave1 | 1082(22%) | 1786(36%) | 2031(41%) |
| Wave2 | 975(20%) | 1536(31%) | 2388(49%) |
| Wave3 | 798(16%) | 1587(32%) | 2514(51%) |
| *First-Generation* | |  |  |
| Wave1 | 147(22%) | 230(34%) | 298(44%) |
| Wave2 | 137(20%) | 200(30%) | 338(50%) |
| Wave3 | 107(16%) | 206(31%) | 362(54%) |
| *Non-Native* |  |  |  |
| Wave1 | 112(21%) | 184(34%) | 240(45%) |
| Wave2 | 107(20%) | 164(31%) | 265(49%) |
| Wave3 | 89(17%) | 165(31%) | 282(53%) |
| **Indigenous** |  |  |  |
| *Non-Indigenous* | |  |  |
| Wave1 | 1262(22%) | 2069(36%) | 2432(42%) |
| Wave2 | 1149(20%) | 1803(31%) | 2811(49%) |
| Wave3 | 948(16%) | 1836(32%) | 2979(52%) |
| *Indigenous* |  |  |  |
| Wave1 | 79(23%) | 131(38%) | 137(39%) |
| Wave2 | 70(20%) | 97(28%) | 180(52%) |
| Wave3 | 46(13%) | 122(35%) | 179(52%) |
