# Supplementary material for: Stability and Instability of Subjective Well-Being in the Transition from Adolescence to Young Adulthood: Longitudinal Evidence from 20991 Young Australians
Source: PLoS One. 2016 May 27;11(5):e0156399. doi: 10.1371/journal.pone.0156399 (PMC4883794; doi:10.1371/journal.pone.0156399)
Supplement: S1 Text — (DOCX) [file pone.0156399.s019.docx]

# S1 Text. Latent Profile Analysis.

***Model Specification***. Adopting the class enumeration method, 1 to 9 classes for latent profile models were first specified. Profile analysis was performed using Mplus 7.1 with MLR estimator. To reduce the probability that the results finalize on the local maximum, we started the estimation with 5000 random starts for each model and 500 best retained for the final optimization. The variances of observed variables were freely estimated across profiles in all the estimated models. Concerns of local maxima was addressed again by examining the results using Mplus TECH11 and TECH14.

***Model Selection***. To determine the number of profiles, following fits statistics were considered: the Akaike Information Criterion (AIC; Akaike, 1987), the Bayesian Information Criterion (BIC; Schwartz, 1978), the Consistent Akaike Information Cariterion (CAIC; Bozdogan, 1987), and Adjusted BIC (ABIC; Sclove, 1987). Lower value of above fit indices suggests better fit. Entropy is another index considered which provides the information how well individuals have been classified into the latent class. It is measured on a scale from zero to one with a value of one indicating individuals are perfectly classified into the latent class (McLachlan & Peel, 2000). LMR test (Lo, Mendel, & Rubin likelihood ratio test) is performed which tests whether k class yields better fit than k-1 class model (Lo et al., 2001). A significant p value confirms current model (k class model) fits better than the k-1 class. BLRT test (Bootstrap Likelihood Ratio Test) is also obtained by bootstrapping when TECH14 is requested in Mplus (McLachlan & Peel, 2000). Rejection of the k-1 class model is obtained with significant p-value. In general, the model comparison does not only rely on the fit criteria, but also on the interpretability of final solution.
