# Supplementary material for: Stability and Instability of Subjective Well-Being in the Transition from Adolescence to Young Adulthood: Longitudinal Evidence from 20991 Young Australians
Source: PLoS One. 2016 May 27;11(5):e0156399. doi: 10.1371/journal.pone.0156399 (PMC4883794; doi:10.1371/journal.pone.0156399)
Supplement: S2 Text — (DOCX) [file pone.0156399.s020.docx]

**S2 Text. Measurement Invariance Test Results.**

We first tested whether the latent construct of life satisfaction measure changes over seven time waves. A complete sequence of measurement invariance test for both samples was conducted and results were presented in Table S3. For Cohort 2003, the fit statistics of all the invariance models tested provided a good fit with CFIs and TLIs greater than .9 and RMSEA values less than .06, the difference in model fit between the more and less constrained model was found to be ignorable and thus yield the conclusion that latent construct we tested is invariant over time. The first step, configural invariance consisted of fitting a three-group (three time waves), three-factor ESEM model to establish the baseline model. The baseline model exhibited good fit characteristics and supports the basic requirement for the further imposition of constrains (χ^2^(378) = 970.391, *p* < 0.01; CFI = 0.994; TLI = 0.991; RMSEA = 0.016). The weak invariance over three time waves was tested constraining the factor loadings of the same items to be identical over time. The estimated weak invariance model fit the data quite well (χ^2^(426) = 1026.624, *p* < 0.01; CFI = 0.994; TLI = 0.992; RMSEA = 0.015), the difference in model fit between the configural invariance and weak invariance is minor (ΔCFI = 0.000; ΔRMSEA = -0.001) and thus the factor loadings relating latent construct and observed variables was equal over time. The next step in the sequence, strong invariance model, holding the threshold equal over time, also fit data well (χ^2^(486) = 1396.514, *p* < 0.01; CFI = 0.990; TLI = 0.989; RMSEA = 0.017). Again, the difference in model fit between the strong invariance and weak invariance is minor (ΔCFI = -0.004; ΔRMSEA = 0.002). Test of strict invariance constraining the uniqueness for each observed variable same over time also shown the same pattern as previous tested models (χ^2^(508) = 1748.875, *p* < 0.01; CFI = 0.987; TLI = 0.986; RMSEA = 0.020; ΔCFI = -0.003; ΔRMSEA = 0.003). Having established the measurement invariance over time, we further tested the structural invariance with variance-covariance matrix and latent mean of factors constrained to be equal subsequently. As shown in Table S3, the structural invariance was accepted. To summarize, the current measurement invariance test provides support for the research question that the existed life satisfaction scale measures the same construct over time. Same results were reported in the analyses on Cohort 1995.
